# Well-being effect of international migration and remittance on human and gender development in South Asian countries

**DOI:** 10.1371/journal.pone.0300597

**Published:** 2024-04-18

**Authors:** Nishad Nasrin, Mohammed Ziaul Haider, Md. Nasif Ahsan

**Affiliations:** Economics Discipline, Social Science School, Khulna University, Khulna, Bangladesh; Public Library of Science, UNITED KINGDOM

## Abstract

This study investigates the well-being effect of international migration and remittance on human and gender development in selected South Asian countries. The study has adopted panel regression analysis using secondary data from the World Development Indicators and United Nations Development Programme. This database contains information on seven South Asian countries from 1995 to 2020. The study simultaneously applied the Levin-Lin-Chu, Breitung and IM-Pesaran unit root tests to check the stationarity of data. After satisfying the condition, econometric models such as Fixed and Random Effects were executed. Pesaran’s test of cross-sectional independence, the Westerlund test for cointegration and VIF tests were performed in order to check the robustness of the results. As a post-diagnostic tool, the Hausman test suggests that the Fixed Effect models are appropriate for each estimation. The results demonstrate that personal remittance positively and significantly affects human and gender development. Similarly, international migration significantly influences human development while negatively affecting gender development. The study suggests that these countries should prioritize attaining higher remittances by sending more international migrants. Similarly, the provision of cheaper formal channels for remitting money and giving incentives can be effective for higher remittance inflow. Moreover, negotiation at the government-to-government level can effectively expand the international labour market of the selected countries.

## Introduction

The new economics of labour migration (NELM) theory argues that households choose migration as a household income diversification strategy. Remittance enriches a household’s capacity to invest more in human capital formation [1, 2]. In contrast, individual-level migration theories, argue that migration decisions and actions are determined by the individual, not the household [3]. Irrespective of decisions and actions, does international migration or remittance contribute to human development? Or can migration or remittance inflow influence a nation’s or household’s well-being? These cutting-edge agendas are still under research and debatable. In the literature, well-being is viewed from two different angles subjective and objective. In general migration researchers define subjective well-being as the quality of life, life satisfaction, and health; meanwhile, objective well-being is mostly through income and investment aspects [4–6]. The well-being effects of migration are almost similar at household and country levels.

Migration contributes to developing discourses through multidimensional socioeconomic issues [7]. The literature argues that remittance inflow enables households and migrant-sending countries to invest in development purposes. For instance, education, health and other socioeconomic infrastructures may contribute to higher human development within a country [8]. Remittance is the outcome of international migration, which acts as a tangible input for the overall development of migrant-sending countries [3, 9–11]. In the literature, both long-term and short-term effects of migration and remittance have been observed from the perspectives of human development and gender development [12–14]. It has been seen from the perspective of different countries and contexts that migration and remittance have mostly positive and significant effects in terms of improving human development index (HDI) score [15–19].

The question is: if migration or remittance positively influences development indicators such as HDI, then to what extent and direction are migration and remittance related to the Gender Development Index (GDI)? In the literature, a mixed outcome is reflected. It is argued that migration and remittance increase disparities; however, in the long run, the effect varies based on the type and pattern of migration history. The effect is more rigorous on poorer communities than on economically well-off communities [20]. This argument suggests that the direction and extent of the impact of migration or remittance on well-being differ based on economic strata, perspective, and migration history. A connection has also been found between migration/remittance and gender development. In terms of achieving sustainable gender development, migration is considered an effective instrument, however, migration action is primarily concentrated on men [21]. Meanwhile, migration contributes to transforming gender roles in the home country [22]. Similarly, remittance-receiving status works as an intrinsic development *mantra* in the home country for issues such as the alleviation of poverty, and changing gender roles [23]. Moreover, migrant women encounter risks and shocks, which triggers them to send more remittances for self-insurance against vulnerabilities [24]. Migration enables women to enjoy freedom of mobility, become involved in economic activities, and make decisions regarding household well-being, which contributes to the gender development of their country [25].

Though literature is available from developing and sab-Saharan African (SSA) countries’ perspectives, little evidence has been found relating to development indicators such as HDI, GDI and gender inequality in the South Asian context. Moreover, the above indicators represent a country’s well-being scenario, viewed through the migration and remittance lens. Furthermore, as relatively recent indicators, gender inequality and gender development have attracted much interest. Against this backdrop, the novelty of this study is its examination of the welfare effects of remittance and migration on human and gender development from the perspective of South Asian countries. The study aims to investigate whether there is any effect of migration/remittance on human and gender development in the context of South Asian countries.

In the successive stage of this manuscript, the study focuses on the literature review with the conceptual framework, materials and methods, results and discussion and finally, concussion and policy recommendations. The materials and methods section provides a glimpse of materials used for data extraction and analysis. The policy recommendations are provided based on the results of the study.

## Literature review

Numerous studies have explored the connection between migration and human development from different countries’ contexts and perspectives. The nexus between remittance and human development has been investigated at the household level and across countries, including sub-Saharan African (SSA) countries, South Asian countries, and other developing and low-income countries [13, 16–19, 25–27]. For analysis, different types of data such as panel, time-series and cross-sectional have been applied in order to examine the effects of remittance and migration on human development; in most cases, the HDI has been used as a proxy for representing human development [13, 16–19, 25, 27–31]. However, the debate continues from different perspectives as the dimensions and definitions of human development vary significantly. The literature argues that migration and remittance have significant positive long-term effects. Interestingly, remittance contributes positively to human development [18, 19, 29, 32, 33]. In Sri Lanka, remittance is a significant predictor of human capital formation. However, the development effect varies based on the government’s approach to migrants, which indicates that institutions and governmental policy are closely intertwined with remittance inflow [32, 34]. A positive association exists between human development and remittance: the composite index combined with long healthy life, knowledge accessibility and a decent living standard [35]. Similarly, the literature argues that a strong connection exists between migration/ remittance and inequality.

The evidence suggests that in South Asia, remittance significantly and positively influences economic growth [36–38]. However, at the household level, worker remittance has a long-term yet minor impact on income inequality [39, 40]. Conversely, a positive relationship between remittance and income inequality at the household level is also evident in the literature. Similarly, the relationship between asset accumulation and income distribution is well described by an inverted U-shaped curve, indicating that effects differ in the short and long term [41, 42]. However, this finding is opposed by Tokhirov et al. [43], who discovered a U-shaped relationship between remittance and income inequality. At the early stage of migration, a community faces higher inequality; however, this declines when migration is a long-term trend. In poorer communities, remittance increases inequality, whereas the reverse effect is observed in more affluent communities [2, 26, 44, 45].

The effects of migration and remittance have sustainable and long-lasting impacts on gender; hence, comprehensive indicators instead of a single indicator should be adopted for analysis [12]. GDI is therefore a frequently used indicator in the literature. The GDI is included as one of the well-being measures however, it needs to be more focused than the HDI. This indicator is often misinterpreted in the literature [13, 14, 46]. GDI exemplifies women’s overall gender well-being and relative health status. It is also argued that compared to other development indicators, GDI is socio-economically a powerful and overarching indicator [47, 48]. Evidence suggests that in economic growth, remittance and GDI have a significant and positive influence [39, 49]. Nevertheless, more research is required in order to understand the dynamic and complex association between migration, remittance and gender development.

After a rigorous literature review, the study found that limited literature is available from different countries and contexts, such as SSA and East Europe. However, literature from the South Asian context is rarely available. Moreover, GDI and HDI are seldom explored with connection to migration and/or remittances. The South Asian countries, especially Bangladesh, India and Pakistan, send a bulk of human resources as labourers to different destinations each year. Therefore, the nexus between migration or remittances with human development and gender development grabs the researcher’s attention more. In this context, this study gains interest to investigate the effects of remittance on gender and human development.

## Materials and methods

### Conceptual understanding

**Fig 1:**
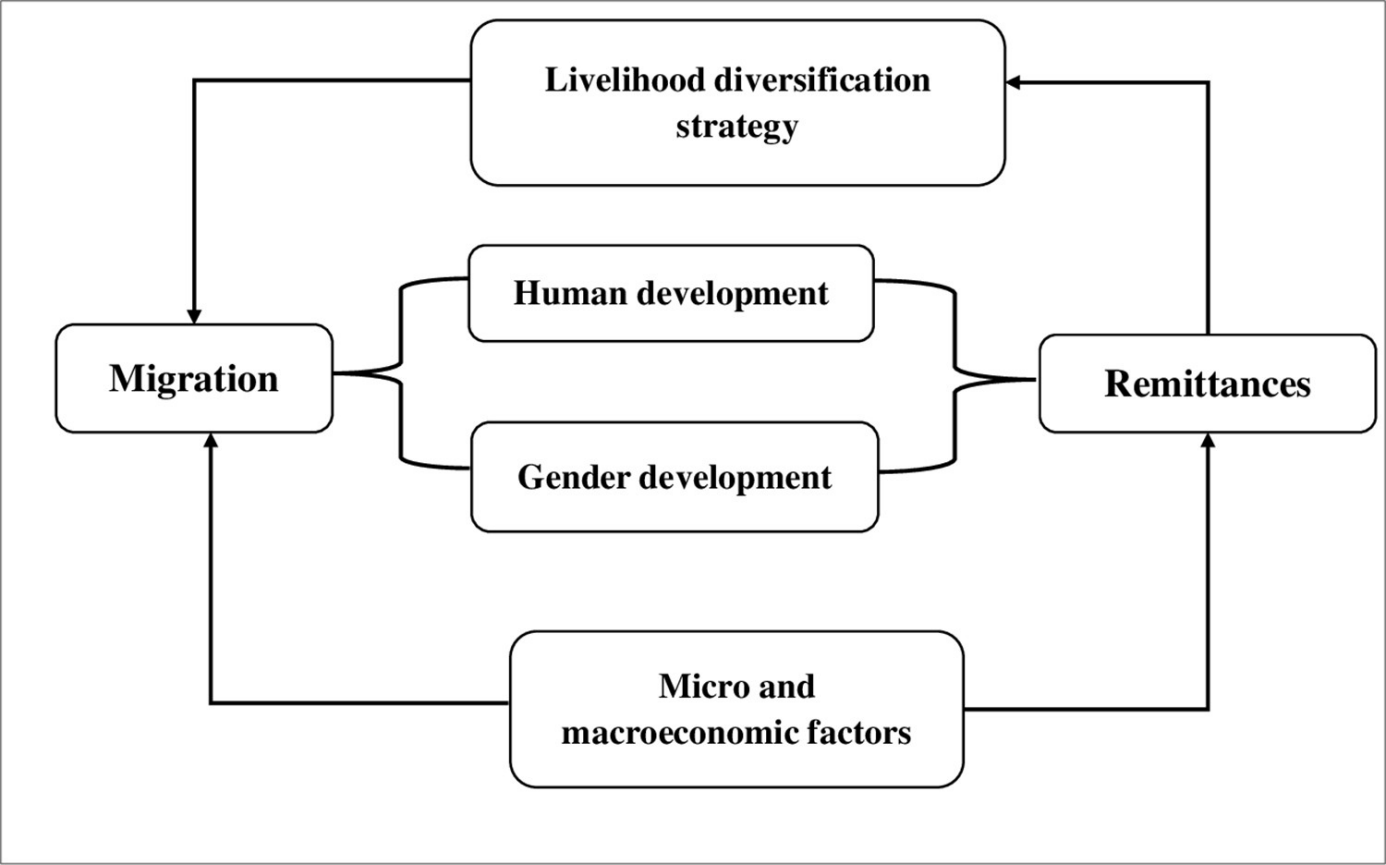
Linkage among migration, remittance, human, and gender development. Source: Adopted from the literature review, 2023.

Migration decision and remittance earning are influenced by a set of micro and also macro economic development indicators where context or circumstances has a predominant role (Fig 1). The literature argues that there is a strong connection between migration/remittance and human development, where the households choose migration as a livelihood diversification strategy.

Similarly, as a outcome of migration, economic and social remittances contributes to uplifting economic growth as well as human and gender development. In this connection, the study aims to explore whether there is any significant influence of migration and remittance on gender and human development in South Asian countries’ context.

### Data

This study adopted panel data in order to estimate the effects of remittance and migration on development indicators. For analysis, the required data were extracted from two database sources: the World Bank (WB) and the United Nations Development Program (UNDP). From these online database sources, data were retrieved between August 25 and 30, 2022. The study focused on seven South Asian countries (Bangladesh, India, Pakistan, Sri Lanka, Maldives, Pakistan and Nepal), while the dataset included a time frame from 1995 to 2020. Bhutan and Afghanistan were removed from the original list of countries because most of the required data for these two countries were missing. Among the variables, personal remittance (in current US$), personal remittance received (% of GDP), international migration stock, and Gini index data were retrieved from World Development Indicators (WDI) [48, 49]. At the same time, data on HDI, GDI, and Gender Inequality Index (GII) were extracted from the UNDP [35]. Data on personal remittance were available for each of the corresponding years. However, international migration stock was calculated accounting for a five-year interval.

Similarly, the countries under study started calculating GDI and GII systematically in 2008; prior to that, the calculation did not follow any systematic pattern. This study used the HDI score as a proxy for human development. The GDI value was considered a proxy for gender development, while the GII value represented gender inequality. This forecasting method balanced the database, i.e., filled up the missing values.

### Dependent variables

#### Human development

A higher index value indicates that a country has moved forward to better health and education levels with higher national income [35]. The components of HDI are:

- a long, healthy life (measured by life expectancy at birth)
- access to knowledge (average and expected schooling years)
- decent living standard (measured by per capita Gross National Income)

### Gender development

Using three fundamental dimensions of human development, the GDI indicates the achievement gaps between women and men [35]. A higher GDI value indicates a better standard of gender development which is expected to be positively linked with remittance. The indicators of GDI are:

- life expectancy at birth
- knowledge (expected and mean schooling years)
- standard of living (GNI per capita) (listed in **Table 1**)

**Table 1:** Variables with descriptive statistics.

Variables	Description	Source	Measurement unit	Expected result	Observations	Mean	Standard deviation	Min	Max
Inequality	The Gini index value is used to represent inequality in income. A higher value shows higher inequality and vice versa.	WDI [50, 51]	Index value	(+/−)	182	35.16	4.99	17.09	44.2
Human development	A higher HDI value shows a higher human development status and vice versa.	UNDP [35]	Index value	(+)	182	0.58	0.10	0.419	0.79
Gender Development	A higher GDI value indicates higher gender development.	UNDP [35]	Index value	(+/−)	182	0.84	0.09	0.598	0.97
Gender Inequality	GII shows a reduction in human development due to differences in achievements between females and males. Sub-indicators are reproductive health (adolescent birth rate and maternal mortality ratio), labour market participation (participation rate of females and males in the labour force), and empowerment (shares of parliamentary seats and population with minimum secondary education by each gender). A higher index value shows a higher inequality.	UNDP [35]	Index value	(−)	182	0.52	0.14	0.014	0.77
Ln (Personal Remittance)	Ln converted personal remittance received (in current US$)	WDI [50, 51]	Percentage (%)	(+)	182	20.25	3.68	11.651	25.15
International Migration Stock	The stock of international migrants is a percentage of the total population.	WDI [50, 51]	Percentage (%) of the total population	(+)	182	3.59	4.23	0.2	14.9

#### Stationarity test

Before regression analysis, unit root tests including Levin-Lin-Chu (LLC) and Breitung unit root tests were performed in order to check whether the series was stationary. In panel estimation, the variables are required to be stationary [52]. Hence, this study used both tests in order to examine whether the variables were stationary at level or at least at first difference. In addition, the IM-Pesaran-Shin test is executed to examine the stationarity of the panels.

#### Levin-Lin-Chu unit root test

For a balanced dataset, the Levin-Lin-Chu unit root test assumes:

$$Null\;Hypothesis:\;H_{0}:\;p_{i}=0_{i}\;\to every\;time\;series\;contains\;unit\;roots$$

$$Alternative\;Hypothesis:\;H_{A}:\;p_{0}=p_{0i}\to every\;time\;series\;is\;stationary$$

Following other unit tests, the LLC also assumes the independence of each cross-section process. The estimated equation below serves as the main foundation for the LLC test.


$$\Delta y_{i,t}=\alpha_{i}+\delta_{it}+\theta_{t}+p_{i}y_{i,\;t-1}+\zeta_{i,t}$$ (1)

In this equation, i = 1, 2, 3, …….N while t = 1, 2, 3………T. The LLC unit root test applied the pooled or augmented Dicky-Fuller test, using various time lags [52].

#### Breitung unit root test

The Breitung unit root test is different from the LLC unit root test. The LLC unit root test depends on *t* statistics of regression analysis which are later revised. The *t*-statistics must be non-zero as it considers panel-specific averages and trends. In perfectly balanced data (a pre-requisite), the Breitung unit root test applies the data-altering technique as an alternative strategy for attaining a *t*-statistic. The t-statistic will be asymptotically generally distributed if T → ∞ and N → ∞. Each of the panels will provide an autoregressive parameter. Likewise, the Levin-Lin-Chu unit root test and the Breitung unit root test also assume:

$$Null\;hypothesis:\;H_{0}:\;Every\;series\;contains\;unit\;roots$$

$$Alternative\;hypothesis:\;H_{A}:\;Every\;series\;is\;stationary$$

The Breitung unit root test best fits when all the panels have the same autoregressive values; however, it is also applicable when heterogeneity exists due to the panel’s parameters. This test has been found to be more powerful and effective for a small number of observations [53, 54].

#### IM-Pesaran-Shin test

The IM-Pesaran-Shin test estimates a t-test in order to examine unit roots in heterogenous panels, it assumes in null hypothesis that all panels are not stationary.


$$Null\;hypothesis:\;H_{0}:\;All\;panels\;contain\;unit\;roots$$

$$Alternative\;hypothesis:\;H_{0}:\;Some\;panels\;are\;stationary$$

#### Empirical, analytical tools

Panel data considers temporal and spatial dimensions, the spatial dimension covers cross-sectional units and the temporal dimension covers periodic observations [55]. This study used panel regression analysis with a panel dataset of seven countries over the period 1995–2020. The *xtreg* command is executed in STATA 17 in regression analysis.

The general formula for panel regression analysis is explained in Eq 2.

$$y_{it}=\alpha_{i}+bx_{it}+{Ɛ}_{it}$$ (2)

Where *α* and *b* are coefficients; *y* indicates dependent variables, *x* indicates explanatory variables; *i* indicates the number of individuals or cross sections, and *t* represents the number of periods. Executing the Fixed Effects (FE) and Random Effects (FE) models is necessary for the panel regression analysis. Following the execution of those models, a diagnostic test is required in order to examine whether the FE or RE model is most appropriate for estimation [56].

#### Fixed Effect (FE) model

The FE model is significantly distinguished from the common and random effect models; nevertheless, it also adopts the basic principles of Ordinary Least Square (OLS) estimates. In FE models, the assumption is that the differences between cross-sections or individuals can be represented using different intercepts. FE models are more reliable than RE models. The Fixed Effect Panel regression equation is provided in Eq (3).

$$y_{it}=\alpha_{i}+\beta^{\prime }X_{it}+{Ɛ}_{it}$$ (3)

Where variables represent the number of cross-sections, i.e., the number of periods, the FE model allows a random variable (individual-specific effect) to be correlated with independent variables [56, 57].

#### Random Effect (RE) model

If the inference variables are likely to be correlated between individuals and periods, then the RE model is generally applied. This model adjusts the difference between intercepts by incorporating error terms. The RE model is more effective in eliminating heteroscedasticity than other models. Hence, this model is also called the Error Component Model (ECM) or Generalized Least Square (GLS) Technique. The FE model holds the basic principles of OLS, in contrast to the RE model. This model contains the principles of GLS or maximum likelihood. The model assumes differences between intercepts for each cross-section or individual; hence, two residual terms exist simultaneously in the equation. Intercept term 1 is a combination of time series and cross-section; intercept term 2 is an individual residual, which is random by nature [56]. The general equation of a RE model is illustrated in Eq 4.


$$y_{it}=\alpha_{i}+\beta^{\prime }X_{it}+u_{i}+{Ɛ}_{it}$$ (iv)

### Post-regression diagnosis–choosing an appropriate model

There are several methods for examining the appropriateness of models, for example, the Hausman test, Chow test, and Test Lagrange Multiplier. This study adopted the Hausman test to test the appropriateness between FE and RE models.


$${Null\;hypothesis:\;H}_{0}=The\;random\;effect\;is\;appropriate$$

$$Alternate\;hypothesis:\;H_{A}=The\;fixed\;effect\;is\;appropriate$$

*Results*: If p>0.05 = RE is appropriate, whereas if p<0.05 = FE is appropriate [56].

*Pesaran’s CD test of cross-sectional independence*. The Pesaran test assumes no cross-sectional dependence under the null hypothesis. Hence, rejection of the null hypothesis will mean that there exists cross-sectional independence.

### Westerlund cointegration test

The Westerlund cointegration test is performed in order to examine whether the panels are cointegrated.


$${Null\;hypothesis:\;H}_{0}=No\;cointegration$$

$${Alternative\;hypothesis:\;H}_{A}=Some\;panels\;are\;cointegrated$$

### Ethical consideration

This study is conducted using secondary sources of data. Hence, no face-to-face interaction was required. Therefore, no sensitive questions were asked or dealt with at any stage of producing the paper.

## Results

### Results of the stationarity test

Before regression analysis, this study examined whether the data was stationary, using LLC and Breitung unit root tests. The results (**Table 2**) from both the tests showed that, personal remittance, personal remittance received (% of GDP), international migration stock, human development, and gender development were significant (1% level) at the first difference. The results also showed that there were no unit roots in the mentioned panels. In contrast, gender inequality did not satisfy the criteria of the LLC test. However, there were significant results when the first difference is considered. While performing the second generation IM-Pesaran-Shin test, the variables were found to be stationary at second difference.

**Table 2:** Results of unit root tests.

Variables (at 2^nd^ difference)	Levin-Lin-Chu	Breitung unit root test	IM-Pesaran-Shin Test
Personal remittance received (% of GDP) Level	**-**0.3155	1.2974	
Personal remittance received (% of GDP) Level (1^st^ difference)	-2.0348**	3.388***	
Personal remittance received (% of GDP) (2^nd^ difference)	-8.8098***	-7.269***	-8.642***
International Migration Stock (Level)	0.7457	7.3846	
International Migration Stock (1^st^ difference)	-1.406**	-2.1304*	
International Migration Stock (2^nd^ difference)	-5.045***	-8.7750***	-6.555***
Inequality (Level)	-1.8741**	4.4543	
Inequality (1^st^ difference)	-1.144	-2.8113***	
Inequality (2^nd^ difference)	-5.8217***	-8.8556***	-6.688***
Human Development (Level)	0.3787	1.8608	
Human Development (1^st^ difference)	-2.362***	-5.339***	
Human Development (2^nd^ difference)	-10.9789***	-9.653***	-8.770***
Gender Development (Level)	-1.2597	3.2973	
Gender Development (1^st^ difference)	-1.4371*	-3.065***	
Gender Development (2^nd^ difference)	-7.6813***	-9.982***	-8.077***
Gender Inequality (Level)	1.7610	7.4342	
Gender Inequality (1^st^ difference)	0.1878	-2.5941**	
Gender Inequality (2^nd^ difference)	-6.229***	-10.027***	-8.296***

Note. Lag in the parenthesis; *** p<0.01

** p<0.05

* p<0.1

### Effects of remittance and migration on human development

The Hausman identification test (1978) (**Table 3**) indicates that the FE model is appropriate for analysing the effects of remittance and migration on human development. The FE panel regression analysis results (**Table 3**) demonstrate that personal remittance has a significant and positive (at a 1% level) effect on human development. A 1% improvement in remittance leads to a positive change in the human development score by 0.00037; this result was expected. Similarly, international migration stock positively affects the HDI score. The results show that if the stock of international migration increases by 1%, the human development score tends to increase by 0.012 points. In addition, one of the vital determinants of human development was found to be negatively connected with gender inequality. It can be seen that if gender inequality increases in terms of achievements (adolescent birth and maternal mortality ratio, labour market participation, and empowerment, i.e., shares in parliament seats, minimum secondary education for each gender), it leads to a decrease in human development score. Conversely, if the gender inequality index increases by 1%, it decreases the human development score by 0.16, which was also expected. The results of Pesaran’s test of cross-sectional independence (**Table 3**) suggest accepting the null hypothesis thus there exists no cross-sectional dependency among the panels. The Westerlund test for checking cointegration also suggests that there exists no cointegration among the panels.

**Table 3:** Effects of remittance and migration on human development (FE).

**DV: Human development**	**Coefficient**	**Standard Error**					**t-value**		**p-value**				**Significance**
Personal remittance received	0.037	0.001						28.35	0.00		***		
International migration stock	0.012	0.002						7.68	0.00		***		
Inequality	-0.001	0.00						-1.08	0.282				
Gender inequality	-0.16	0.018						-9.02	0.00		***		
Constant	-0.115	0.037						-3.06	0.003		***		
Mean dependent variable	0.579		SD dependent variable									0.098	
R-squared	0.879		Number of observations									182	
F-test	311.198		Prob > F									0.000	
Akaike criteria (AIC)	-907.676		Bayesian criteria (BIC)									-891.656	
***Hausman Specification Test (1978)***													
*Null Hypothesis*: *H*_*0*_ *= The random effect is appropriate* *Alternative Hypothesis*: *H*_*a*_ *= The fixed effect is appropriate*													
Hausman Test								*χ*^*2*^ *= 60*.*82*		*p-value*: *0*.*00*			
**Decision:** The fixed effect is appropriate													
**Pesaran’s test of cross-sectional independence**								*= 3*.*815*		*p-value*: *= 0*.*113*			
***Decision***: Cross-sectional independence													
**Westerlund test for cointegration:**													
*Null Hypothesis*: H0 = No cointegration *Alternative Hypothesis*: Ha = Some panels are cointegrated					*p-value*: *0*.*3159*								
***Decision*:** Panels are not cointegration.													
**VIF test:**				2.83									

Note. Lag in the parenthesis; *** p<0.01

** p<0.05

* p<0.1

### Remittance and migration–gender development lens

For this model, the Hausman specification test suggests the FE model is appropriate (**Table 4**). If remittance increases by 1%, then the GDI score also increases by 0.00019 points (significant at a 1% level), and the sign and direction are as expected. The results also portray that if international migration increases by 1%, the GDI score tends to decline by 0.004 points. As expected, a 1 unit increase in the HDI score leads to an improvement in gender development by 0.2 points (significant at a 1% level). The result suggests that if the overall HDI score increases, the gap between women and men reduces. Moreover, the GII is inversely related to the GDI i.e., if the GII value rises by 1 point, the GDI declines by 0.119 points. GII demonstrates a reduction in HDI based on differences in achievements between women and men in terms of reproductive health, labour market participation, and empowerment. The Pesaran’s test of cross-sectional independence demonstrates that the cross-section panels are independent, while the Westerlund test for cointegration suggests that the panels are not-cointegrated.

**Table 4:** Effects of remittance and migration on gender development (FE).

**DV: Gender Development**	**Coefficient**						**Standard Error**						**t-value**		**p-value**	**Significance**
Personal remittance received					0.019		0.002				7.77				0.00	***
International migration stock			-0.004				0.001				-2.85				0.005	***
Inequality	0.00						0.00				1.04				0.299	
Human development		0.279					0.06				4.66				0.00	***
Gender inequality	-0.119						0.017				-7.09				0.00	***
Constant	0.35						0.03				11.62				0.00	***
Mean dependent variable	0.835					SD dependent variable								0.092		
R-squared	0.877					Number of observations								182		
F-test	242.686					Prob > F								0.000		
Akaike criteria (AIC)	-995.879					Bayesian criteria (BIC)								-976.655		
***Hausman Specification Test (1978)***																
*Null Hypothesis*: *H*_*0*_ *= The random effect is appropriate* *Alternative Hypothesis*: *H*_*a*_ *= The fixed effect is appropriate*																
Hausman Test				χ^2^ = 172.579						*p*-value: 0.00						
***Decision*:** The fixed effect is appropriate																
**Pesaran’s test of cross-sectional independence:**								= -2.477				*p*-value *= 0*.*132*				
***Decision***: Cross-sectional independence																
**Westerlund test for cointegration:**																
*Null Hypothesis*: H0 = No cointegration *Alternative Hypothesis*: Ha = Some panels are cointegrated										*p*-value: *0*.*1167*						
***Decision*:** Panels are not cointegrated																
**VIF test**									*4*.*16*							

Note. Lag in the parenthesis; *** p<0.01, ** p<0.05, * p<0.1

## Discussion

### Remittance, migration and human development

This study found that remittance positively and significantly contributes to the human development score with the expected sign and direction. This finding is consistent with the existing literature [19, 28, 34]. Workers’ international remittance positively affects human development in the long run. Hence, remittance acts as the backbone of many developing and sab-Saharan African countries [27, 29, 31]. The HDI indicates the overall quality of life of a country’s people, which is significantly and positively enhanced by the remittance inflow [27]. It can be understood that with a higher remittance inflow, the economy also benefits; this is because, with the heightened remittance, country’s capability to invest in development sectors increase. This investment in direct productive and human development sectors, such as health and education, results in a positive move towards human development. With higher investment of remitted money, the country can move forward to attain a longer life expectancy.

Similarly, this investment enables countries to achieve higher average years of schooling. These two indicators of human development directly improve a country’s human capital formation, which will help to safeguard future national income through better livelihood opportunities [58]. Remittance also helps to achieve a decent standard of living by increasing per capita Gross National Income (GNI). This finding was also expected because Bangladeshi migrants’ income is added to the national accounts. Moreover, the remitted money is primarily invested in productive activities, which may raise national income.

Likewise, international migration stock also positively affects HDI score. However, this finding contradicts the findings of Akanbi [30], who reported a negative effect of migration on human development in the African context. Likewise, Sanderson and Kentor [30] also reported a negative effect of migration on human development from developing countries’ perspectives. Migration brings not just economic remittances but also social remittances, such as attitude, knowledge, beliefs, practices etc. [30]. This may indirectly influence socioeconomic and cultural development, leading to higher human development scores. Migration is not only essential for earning foreign currency but also contributes to socioeconomic and cultural transformation.

The study also found that human development is associated with lower gender inequality. Lowering inequality between women and men can ensure the sustainable development of a country [35, 59]. The results allowing women to fall behind men will not lead to positive growth in human development. In South Asian countries, women lag behind in health, labour market participation, and empowerment dimensions. In South Asia, by adopting the quota system, Bangladesh has allotted 13% of it’s government seats, Pakistan 17% of seats in its national assembly, and India 8.3% of seats in the lower house to women [60]. Through alloting more seats to women in parliaments, increasing access to labour market participation, and providing better health service for women, gender gaps in achievement between women and men can be lessened.

### Remittance, migration and gender development

Among different socioeconomic indicators, gender plays a critical role in sustainable development. It is also evident in the agro-climatic zones in Pakistan, where poverty is predominant with other socioeconomic vulnerabilities. Poverty reduction as a development indicator is also influenced by a number of determinants. Increased inflow of FDI also stimulates employment and economic growth [37, 61–65].

In this study, the findings suggest that in the south Asian countries, remittance is positively associated with gender development. This may be because higher remittance can be invested in women’s development, such as education and health, which can increase life expectancy at birth. In addition, through remittance, infrastructure for access to education can be attained, which may lead to an increase in expected and average years of schooling. Investment in the productive sector can also heighten national income, including a higher share of women’s contribution, which may lead to better living standards [36–38]. Remittance also improves economic well-being, including better dietary intake and health status for women [66]. Moreover, higher educational investments promote women’s education [67]. The literature argues that remittance nurtures socioeconomic development, such as an increased standard of living and sustainability [67].

Conversely, international migration leads to a decrease in gender development. This may happen because the migration rate of women is significantly lower than that of men in South Asian countries such as Bangladesh [68]. In a developing country, generally, men choose migration as a livelihood diversification strategy, while women are left behind to tend to household responsibilities. They are often restricted by other household members from going outside for education or healthcare services. Moreover, remittance expenditure depends on gender, type of family, and household head [69–71]. Likewise, households in a country may not benefit if the country’s migration is male-centric. Mobilizing women towards international migration can be an effective tactic for women’s development. Similarly, circumstances such as the location of a household have an effective on international migration belongs. Rural households face socioeconomic and cultural bottlenecks and other obstacles to women’s development. Gender development may also depend on good governance, institutional coordination, gender-centric policies, and effective implementation of policies.

In contrast, human development is positively related to gender development. If the overall HDI score increases, the gap between women and men may reduce, which implies that gender development is ensured in terms of achievement in life expectancy, knowledge, and GNI per capita. Conversely, GII demonstrates a reduction in HDI based on differences in achievements between women and men regarding reproductive health, labour market participation, and empowerment. If the adolescent birth rate and maternal mortality ratio increase, if labour force participation declines, if the share of women in parliament declines; and if the minimum population with secondary education declines, GDI will be negatively affected. In other words, if gender inequality decreases, it indicates that gender is developing, i.e., inequality between women and men is decreasing.

## Strengths and limitations

This study has some unique strengths; it examines the effects of remittance on human development and gender in the South Asian context, which adds value to the existing literature. In the South Asian context effects of remittance on macroeconomic determinants have been relatively less often explored in the literature. Furthermore, little evidence is found reagrding the nexus between remittance on gender development indicators which is also investigated in this study. This paper has some limitations, such as data scarcity on the stock of international migration. International migration stock data is available at five-year intervals. In addition, data on GDI and GII were not collected systematically for each country and each year. The study focused on seven countries in South Asia belonging to SAARC and covered 26 years of data starting from 1995. Countries such as Afghanistan and Bhutan were removed from the list due to the unavailability of required data. Databases covering more countries and years could provide more robust results. Along with a regional scenario of migration and well-being, country-specific analysis can provide a detailed overview of the effects of remittance and migration.

### Policy recommendations

The results demonstrate that migration stock and remittance are two significant predictors of human development; hence, policies like extending migration stock towards different lucrative destinations that provide higher wages and job securities should be prioritised. Instead of sending unskilled labourers, the countries should prioritize sending skilled labourers abroad to get higher remittance from the destination countries. In this regard, in collaboration with NGOs, the government may launch different skill development initiatives like training and motivational sessions may be effective. Similarly, to increase remittances through formal channel, incentives to the sending amount may be fruitfull. In addition, the migrant sending countries should explore and extend new labour markets for sustainable development.

## Conclusion

The literature argues that a connection exists between migration/remittance and development indicators such as human and gender development. In the literature, the association has been measured from different countries’ contexts and perspectives; however, few studies have investigated the connection from the perspective of South Asian countries. The novelty of this study lies in its examination of the effects of migration and remittance on human development and gender development from this perspective. The study found that human development, remittance, and stock of international migration positively and significantly affect human development [36, 62, 64]; however, gender inequality negatively affects human development. The findings confirm that in human development, both remittance and international migration stock have an influential positive role; hence, interventions should be undertaken to increase remittance earnings through a higher stock of international migrants in order to achieve sustainable development. Likewise, on gender development, remittance influences positively; however, international migrant stock affects it negatively. This result was expected, as there are still different dynamics in gender development due to migration. Inequality reduction, gender development, higher remittance, and migration stock play a crucial role for sustainable human development [38]; therefore, policies for increasing remittance inflow by sending more international migrants can be effective. In the South Asian context, priory should be given to the skills development of the young people through motivational and training sessions. In collaboration with non-government organizations, the government could move forward in this regard. Moreover, incentive provisions for increasing remittance could also be effective. Searching for new labour markets and negotiating with the government could also create better opportunities for international job seekers. Therefore, a higher scope of earning remittance would be opened up. To encourage more international migration, migration-friendly policies such as training for skills development, workshops, and seminars could be arranged in order to stimulate awareness among the younger generation. Finally, host countries should encourage skilled workers to make migration decisions for higher remittance attainment. This migration strategy should not be confined to men; women should also be encouraged to migrate internationally.

## References

[1] Massey DS, Arango J, Hugo G, Kouaouci A, Pellegrino A, Taylor JE. Theories of international migration: A review and appraisal. Popul Dev Rev 1993;19:431–66.

[2] Stark O, Bloom DE. The new economics of labor migration. Am Econ Rev 1985;75:173–8.

[3] Castles S, Miller MJ, Ammendola G. The age of migration: International population movements in the modern world. Am Foreign Policy Interes 2005;27:537–42. 10.1080/10803920500434037.

[4] O’Connor KJ. The effect of immigration on natives’ well-being in the European Union. J Econ Behav Organ 2020;180:257–74. 10.1016/j.jebo.2020.10.006.

[5] Manfred M. Fischer PN, editor. Handbook of regional science. 2nd ed. Springer Berlin, Heidelberg; 2021. 10.1007/978-3-662-60723-7.

[6] Western M, Tomaszewski W. Subjective wellbeing, objective wellbeing and inequality in Australia. PLoS One 2016;11:1–20. doi: 10.1371/journal.pone.0163345 27695042 PMC5047468

[7] De Haas H. Migration and development: A theoretical perspective. Int Migr Rev 2010;44:227–64. doi: 10.1111/j.1747-7379.2009.00804.x 26900199 PMC4744987

[8] Sikder MJU, Ballis PH. Remittances and life chances: A study of migrant households in rural Bangladesh. Migr Dev 2013;2:261–85. 10.1080/21632324.2013.814322.

[9] Skeldon R. Migration and development. United Nations Expert Gr. Meet. Int. migation Dev. Asia pacific, Bangkok, Thailand: 2008, p. 09.

[10] Raihan S., Uddin SA. Bangladesh. Migr. Remit. Dev. South Asia, 2011. doi: 10.4135/9788132107842

[11] Nasrin N, Haider MZ, Ahsan MN. Migration As an Adaptation Strategy: a Bibliometric Analysis. Khulna Univ Stud 2022:690–701. 10.53808/kus.2022.icstem4ir.0133-se.

[12] Bachan A. An exploration of the gender-migration-development nexus: The impact of labor migration on women’s empowerment. Cons J Sustain Dev 2018;20:1–22.

[13] Schüler D. The uses and misuses of the gender‐related development index and gender empowerment measure: A review of the literature. J Hum Dev 2006;7:161–81. 10.1080/14649880600768496.

[14] KC BK. Migration, poverty and development in Nepal. Asian Pacific Migr J 2004;13:205–32. 10.1177/011719680401300204.

[15] Wu J, Kilby P. The precarity of gender, migration, and locations: case studies from Bangladesh and Nepal. Dev Pract 2022;33:145–55. 10.1080/09614524.2022.2057441.

[16] Arshad M, Abbas F, Kächele H, Mehmood Y, Mahmood N, Mueller K. Analyzing the impact of government social spending, population growth and foreign remittances on human development in Pakistan: Implications for policy. Eur J Dev Res 2022;34:1607–26. 10.1057/s41287-021-00435-8.

[17] Imran K, Devadason ES, Kee Cheok C. Developmental impacts of remittances on migrant-sending households: Micro-level evidence from Punjab, Pakistan. J South Asian Dev 2019;14:338–66. 10.1177/0973174119887302.

[18] Kamalu K, Ibrahim WHBW. International remittances and human development in developing countries: A panel quantile regression via moment approach. Stud Appl Econ 2022;40. 10.25115/eea.v40i1.5577.

[19] Huay CS, Winterton J, Bani Y, Matemilola BT. Do remittances promote human development? Empirical evidence from developing countries. Int J Soc Econ 2019;46:1173–85. 10.1108/IJSE-12-2018-0673.

[20] Maharjan A, Bauer S, Knerr B. Do rural women who stay behind benefit from male out-migration? a case study in the hills of Nepal. Gend Technol Dev 2012;16:95–123. 10.1177/097185241101600105.

[21] Holliday J, Hennebry J, Gammage S. Achieving the sustainable development goals: surfacing the role for a gender analytic of migration. J Ethn Migr Stud 2019;45:2551–65. 10.1080/1369183X.2018.1456720.

[22] Lopez-Ekra S., Aghazarm C., Kötter H., Mollard B. The impact of remittances on gender roles and opportunities for children in recipient families: research from the International Organization for Migration. Gend Dev 2011;19:69–80. 10.1080/13552074.2011.554025.

[23] Van Naerssen T, Smith L, Davids T MM. Women, gender and remittances: An introduction. In: Ton van Naerssen, Smith Lothar, Tine Davids MMHM, editor. Women, gender, Remit. Dev. Glob. South, England: Ashgate Publishing, Limited; 2015, p. 5–13.

[24] Goff ML. Feminization of migration and trends in remittances. IZA World Labor 2016.

[25] Ullah AKMA. Male migration and ‘left–behind’ women: Bane or boon? Environ Urban ASIA 2017;8:59–73. 10.1177/0975425316683862.

[26] Gonzalez-Konig G., Wodon Q. Remittances and inequality. 2005.

[27] Deluna RJ, Pedida S. Overseas Filipino workers remittances, inequality and quality of life in the Philippines. Munich Pers RePEc Arch 2014;56070.

[28] Kausar F, Sarwar S, Rafiq MY, Ali R, Rehman RU. Integrating migration, human development and remittances: an analysis of SAARC countries. Int J Happiness Dev 2019;5:115. 10.1504/ijhd.2019.099354.

[29] Adenutsi D. Long-run macroeconomic impact of international migrant remittances on human development in low-income countries: A panel analysis of sub-saharan Africa. J Int Econ Stud 2010;24:113–32.

[30] Akanbi OA. Impact of migration on economic growth and human development: Case of Sub-Saharan African countries. Int J Soc Econ 2017;44:683–95. 10.1108/IJSE-07-2015-0190.

[31] Naeem MZ, Arzu S. The role of remittances on human development: Evidence from developing countries. Bull Bus Econ 2017;6:74–91.

[32] Ustubici A, Irdam D. The impact of remittances on human development: A quantitative analysis and policy implications. Econ Sociol 2012;5:74–95. 10.14254/2071-789X.2012/5-1/6.

[33] Mohammed U. Remittances, institutions and human development in Sub-Saharan Africa. J Econ Dev 2022;24:142–57. 10.1108/jed-03-2021-0041.

[34] Aslam ALM, Sivarajasingham S. Annual international research conference. Do Work. Remit. Promot. Hum. Cap. Form. Sri Lanka?, Sri Lanka: Faculty of Management and Commerce; 2019, p. 92–105.

[35] UNDP (United Nations Development Programme). Human Development Reports 2022. https://hdr.undp.org/data-center/specific-country-data#/countries/SGP.

[36] Wang Z, Zaman S, Zaman QU RS. Impact of remittances on carbon emission: Fresh evidence from a panel of five remittance-receiving countries. Environ Sci Pollut Res 2021;28:52418–52430. doi: 10.1007/s11356-021-14412-5 34008066

[37] Khan I., Xue J., Zaman S., Mehmood Z. Nexus Between FDI, Economic Growth, Industrialization, and Employment Opportunities: Empirical Evidence from Pakistan. J Knowl Econ 2023;14:3153–3175. 10.1007/s13132-022-01006-w.

[38] Shah Z, Zilong W, Qamar UZ. Exploring the relationship between remittances received, education expenditures, energy use, income, poverty, and economic growth: fresh empirical evidence in the context of selected remittances receiving countries. Environ Sci Pollut Res 2021;28:17865–17877. 10.1007/s11356-020-11943-1. 33400110

[39] Islam MS. Do personal remittances influence economic growth in South Asia? A panel analysis. Rev Dev Econ 2022;26:242–58. 10.1111/rode.12842.

[40] Taylor JE. Remittances and inequality reconsidered: Direct, indirect, and intertemporal effects. J Policy Model 1992;14:187–208. 10.1016/0161-8938(92)90008-Z.

[41] Barham B, Boucher S. Migration, remittances, and inequality: Estimating the net effects of migration on income distribution. J Dev Econ 1998;55:307–31. doi: 10.1016/s0304-3878(98)90038-4 12293843

[42] Shen IL, Docquier F, Rapoport H. Remittances and inequality: A dynamic migration model. J Econ Inequal 2010;8:197–220. 10.1007/s10888-009-9110-y.

[43] Tokhirov A, Harmáček J, Syrovátka M. Remittances and inequality: The post-communist region. Prague Econ Pap 2021;30:426–48. 10.18267/j.pep.776.

[44] Giannetti M, Federici D, Raitano M. Migrant remittances and inequality in Central-Eastern Europe. Int Rev Appl Econ 2009;23:289–307. 10.1080/02692170902811710.

[45] Rapoport H, Docquier F. The economics of migrants? Remittances. 2005.

[46] Pankaj A, Tankha R. Empowerment effects of the NREGS on women workers: A study in four states. Econ Polit Wkly 2010;45:45–55.

[47] Lopez-Claros A., Zahidi S. F économique mondial. Women’s empowerment: Measuring the global gender gap. Forum économique Mond., 2005, p. 91–3.

[48] Folbre N. Measuring care: Gender, empowerment, and the care economy. J Hum Dev 2006;7:183–99. 10.1080/14649880600768512.

[49] Oniyke S.C., Ekeagwu I.C. ACS. International remittances and economic growth of Nigeria. AE-Funai J Accounting, Bus Financ 2020;6:152–66.

[50] WDI (World Development Indicators). World Bank data portal 2022. https://www.migrationdataportal.org/international-data?i=stock_perc_&t=2020&m=1.

[51] WDI (World Development Indicators). World Bank data portal 2022. https://databank.worldbank.org/source/world-development-indicators.

[52] Bekun FV. Mitigating emissions in India: Accounting for the role of real income, renewable energy consumption and investment in energy. Int J Energy Econ Policy 2022;12:188–92. 10.32479/ijeep.12652.

[53] Breitung J. The local power of some unit root tests for panel data. Nonstationary panels, panel cointegration, Dyn. panels, Emerald Group Publishing Limited; 2001, p. 161–77.

[54] Breitung J, Das S. Panel unit root tests under cross-sectional dependence. Stat Neerl 2005;59:414–33. 10.1111/j.1467-9574.2005.00299.x.

[55] Yaffee R. A primer for panel data analysis. Connect Inf Technol NYU 2003;8:1–11.

[56] Zulfikar R. Estimation model and selection method of panel data regression: An overview of common effect, fixed effect, and random effect model. J Ilm Bid Akunt 2018:1–18.

[57] Schmidheiny K, Unversität B. Panel data: fixed and random effects. Short Guid to Microeconometrics 2011;7:2–7.

[58] Ellis F. Household strategies and rural livelihood diversification. J Dev Stud 1998;35:1–38. doi: 10.1080/00220389808422553

[59] Lopez-Claros, Augusto SZ. Women’s empowerment: Measuring the global gender gap, Geneva: World Economic Forum; 2005, p. 91–3.

[60] Rai SM. Reserved seats in South Asia: A regional perspective. In:: Julie Ballington AK, editor. Parliam Women. Beyond numbers, Stockholm, Sweden: International Institute for Democracy and Electoral Assistance (IDEA); 2005, p. 205–26. 10.4324/9780203186275-13.

[61] Zaman S, uz Zaman Q, Zhang L, Wang Z, Jehan N. Interaction between agricultural production, female employment, renewable energy, and environmental quality: Policy directions in context of developing economies. Renew Energy 2022;186:288–98. 10.1016/j.renene.2021.12.131.

[62] Wang Z, Uz Zaman Q, Zaman S. Dynamical assessment of multidimensional poverty in agro-climatic zones: an evidence from Punjab Pakistan. Environ Sci Pollut Res 2021;28:22944–22956. 10.1007/s11356-020-12329-z. 33432413

[63] Uz Zaman Q, Zhao Y, Zaman S, Marim A, Jehan N. Spatial evaluation of multidimensional energy poverty between farming and non-farming communities of agro-climatic zones of Pakistan. Energy Policy 2023;172:113294. doi: 10.1016/j.enpol.2022.113294

[64] Honglan J, Shah Z, Uz Zaman Q, Aadil Hameed S, Jie L. A pathway to a sustainable future: Investigating the contribution of technological innovations, clean energy, and Women’s empowerment in mitigating global environmental challenges. J Clean Prod 2023;421:138499. 10.1016/j.jclepro.2023.138499.

[65] Uz Zaman Q, Zhao Y, Zaman S, Aadil Hameed S. Examining the symmetrical effect of traditional energy resources, industrial production, and poverty lessening on ecological sustainability: Policy track in the milieu of five neighboring Asian economies. Resour Policy 2023;83:103606. 10.1016/j.resourpol.2023.103606.

[66] Lu Y. Household migration, social support, and psychosocial health: The perspective from migrant-sending areas. Soc Sci Med 2012;74:135–42. doi: 10.1016/j.socscimed.2011.10.020 22169626 PMC3259452

[67] Azizi SS. The impacts of workers’ remittances on poverty and inequality in developing countries. Empir Econ 2021;60:969–91. 10.1007/s00181-019-01764-8.

[68] BMET (Bangladesh Bureau of Manpower Employment and Training). Oversas employment of female workers (country-wise) from 1991 to 2021 2022. http://www.old.bmet.gov.bd/BMET/statisticalDataAction.

[69] Fakir AMS, Abedin N. Empowered by absence: Does male out-migration empower female household heads left behind? J Int Migr Integr 2021;22:503–27. doi: 10.1007/s12134-019-00754-0

[70] Desai S, Banerji M. Negotiated identities: Male migration and left-behind wives in India. J Popul Res 2008;25:337–55. doi: 10.1007/BF03033894 20694050 PMC2916725

[71] Arokkiaraj H, Kaushik A, Rajan SI. Effects of international male migration on wives left behind in rural Tamil Nadu. Indian J Gend Stud 2021;28:228–47.

